# The benefit of preventing exposure keratopathy in icu patient: a case report

**DOI:** 10.11604/pamj.2016.23.59.4932

**Published:** 2016-02-29

**Authors:** Adil Belmokhtar, Rajae Daoudi

**Affiliations:** 1 Université Mohammed V Souissi, Service d'Ophtalmologie A de l'Hôpital des Spécialités, Centre Hospitalier Universitaire, Rabat, Maroc

**Keywords:** Keratopathy, icu patient, acoustic neuroma

## Image in medicine

We report the case of 55 year old man hospitalized in intensive care unit following a complication of his surgery for acoustic neuroma, for which he was intubated. During his hospitalization, he presented a bilateral exposure keratitis complicated by an abscess and corneal perforation. The ocular surface is protected by the tear film, the blinking of eyelids and the lid closure. The tear film provides lubrication of the cornea and also contains antimicrobial substances. Use of muscle relaxants and sedation in patients on ventilator contributes to inadequate lid closure by decreasing the tonic contraction of ocular muscles. The constant exposure of the ocular surface put the ICU patients at high risk of developing exposure keratopathy. This condition predisposes to microbial keratitis, which may lead to corneal perforation and visual loss. Previous studies have reported that about 40% of patients develop exposure keratopathy during their stay in the ICU. The use of ocular lubricants and securing tape over the eyelids in intubated patients can prevented it. Also, the use of swimming goggles and regular moistening of eyelids providing a moisture chamber could be more effective.

**Figure 1:**
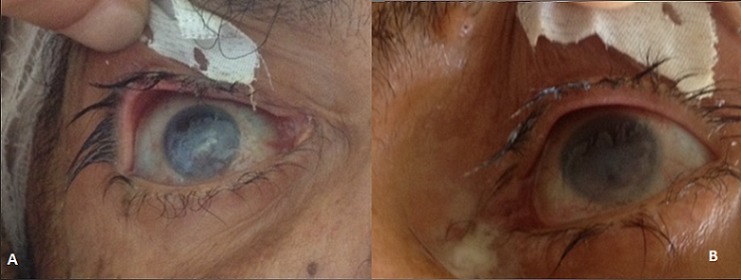
Bilateral exposure keratitis complicated by an abscess and corneal perforation. (A) right eye; (B) left eye

